# Combined UV-C Technologies to Improve Safety and Quality of Fish and Meat Products: A Systematic Review

**DOI:** 10.3390/foods12101961

**Published:** 2023-05-11

**Authors:** Maria Lúcia Guerra Monteiro, Yhan da Silva Mutz, Karen de Abreu Francisco, Denes Kaic Alves do Rosário, Carlos Adam Conte-Junior

**Affiliations:** 1 Graduate Program in Food Science (PPGCAL), Institute of Chemistry (IQ), Federal University of Rio de Janeiro (UFRJ), Cidade Universitária, Rio de Janeiro 21941-909, RJ, Brazil; yhan.mutz@ufrj.br (Y.d.S.M.); karenabreu98@gmail.com (K.d.A.F.); carlosconte@hotmail.com (C.A.C.-J.); 2 Center for Food Analysis (NAL), Technological Development Support Laboratory (LADETEC), Federal University of Rio de Janeiro (UFRJ), Cidade Universitária, Rio de Janeiro 21941-598, RJ, Brazil; 3 Laboratory of Advanced Analysis in Biochemistry and Molecular Biology (LAABBM), Department of Biochemistry, Federal University of Rio de Janeiro (UFRJ), Cidade Universitária, Rio de Janeiro 21941-909, RJ, Brazil; 4 Graduate Program in Veterinary Hygiene (PPGHV), Faculty of Veterinary Medicine, Fluminense Federal University (UFF), Vital Brazil Filho, Niterói 24220-000, RJ, Brazil; 5 Center for Agrarian Sciences and Engineering, Federal University of Espírito Santo (UFES), Alto Universitário, S/N Guararema, Alegre 29500-000, ES, Brazil; deneskaic@gmail.com; 6 Graduate Program in Chemistry (PGQu), Institute of Chemistry (IQ), Federal University of Rio de Janeiro (UFRJ), Cidade Universitária, Rio de Janeiro 21941-909, RJ, Brazil

**Keywords:** UV-C radiation, hurdle technology, decontamination, shelf life, oxidative stability, sensory attributes

## Abstract

This study aimed to identify the best UV-C combined treatments for ensuring the safety and quality of fish and meat products. A total of 4592 articles were screened in the relevant databases, and 16 were eligible studies. For fish, the most effective treatments to reduce Gram-negative and Gram-positive bacteria were UV-C at 0.5 J/cm^2^ + non-thermal atmospheric plasma (NTAP) for 8 min (33.83%) and 1% Verdad N6 + 0.05 J/cm^2^ + vacuum packaging (25.81%), respectively. An oxygen absorber with 0.102 J/cm^2^ was the best combined treatment, reducing lipid oxidation (65.59%), protein oxidation (48.95), color (ΔE = 4.51), and hardness changes (18.61%), in addition to a shelf-life extension of at least 2 days. For meat products, Gram-negative bacteria were more reduced by nir-infrared heating (NIR-H; 200.36 µW/cm^2^/nm) combined with 0.13 J/cm^2^ (70.82%) and 0.11 J/cm^2^ (52.09%). While Gram-positive bacteria by 0.13 J/cm^2^ with NIR-H (200.36 µW/cm^2^/nm), 1, 2, or 4 J/cm^2^ with flash pasteurization (FP) during 1.5 or 3 s, and 2 J/cm^2^ with FP for 0.75 s (58.89–67.77%). LAE (5%) + 0.5 J/cm^2^ was promising for maintaining color and texture. UV-C combined technologies seem to be a cost-effective alternative to ensure safety with little to no quality changes in fish and meat products.

## 1. Introduction

Fish and meat products are rich in essential nutrients (protein, unsaturated fatty acids, and vitamins) for the human diet and are widely consumed worldwide [1,2]. According to the OECD/FAO [3] and FAO [2], the world’s fish and meat production chains have been growing increasingly in the last six decades, mainly due to increasing population growth demanding high-value animal protein. Simultaneously, consumer demand for high-quality products with no chemical preservatives is continuously increasing. 

Despite these facts, fish and meat products are highly perishable due to their intrinsic properties, such as water activity, pH, high protein, and unsaturated fatty acid levels, which are favorable for the growth of microorganisms and oxidative degradation, accelerating negative changes in color, texture, and flavor, consequently resulting in a rapid loss of quality and short shelf life [4,5,6]. According to the Food and Agriculture Organization of the United Nations, 21% of all meat produced globally is discarded by retail markets and consumers due to loss of quality, leading to significant economic losses [7]. For fish, 30–35% is discarded worldwide from catch to final consumption [2]. Furthermore, fish and meat products represent a potential vehicle for pathogens commonly involved in outbreaks, especially those that are consumed raw or undercooked [8]. According to the Center for Disease Control and Prevention of the United States [8], fish (37.17%), chicken (23.11%), and beef (19.9%) were the most outbreak-associated foods in 2017. Aware of these facts, the FAO has encouraged studies focusing on emerging preservation technologies to ensure food safety and extend the shelf life without compromising the food’s original quality parameters, aiming to reduce economic losses and enhance the fish and meat production chains [7].

UV-C light at approximately 260 nm is an emerging non-thermal technology approved for application in food and food products [9]. UV-C light has been studied for its effectiveness against microorganisms through direct action on the microbial DNA and indirect action through free radicals from the radiolysis of water [10,11]. Moreover, UV-C light has several advantages, making it promising to optimize the meat production chain: for example, it is easy to install and apply with no need to change the food production flow, it does not produce toxic residues, it is applied to packaged products avoiding recontamination, it has low cost, and saves energy due to short treatment times [10,12]. Another great advantage of UV-C light is the possibility of causing no or minimal physicochemical and sensory changes, depending mainly on the UV-C dose, food composition, and aspects related to food microbiota (initial load and type of microorganisms) [13,14,15,16,17]. UV-C has been commercially applied for decontaminating water/ice, packaging materials, and liquid products, such as juices. Regarding UV-C equipment cost, it depends especially on the number and irradiance power of the lamps, but, in general, UV-C lamps are inexpensive. In a comparative study, the average energy consumption and cost of heat and UV-C treatments were very similar [11].

However, UV-C light has low penetration power and, in general, doses that reach their antimicrobial effect induce oxidative degradation, impairing the overall meat quality [18,19], which represents the main limitation of their industrial use in foods. Nevertheless, considering its low cost, combining UV-C light with other preservation methods (e.g., high hydrostatic pressure, flash pasteurization, non-thermal atmospheric plasma, nir-infrared heating, modified atmosphere packaging, and oxygen scavengers) could be a key way to enable this industrial preservation technology.

So far, combined UV-C light with conventional and emerging technologies has been successfully applied for single quality parameters (pathogen reduction/inactivation, bacterial growth, physicochemical parameters, or sensory attributes) in fish and meat products [5,20]. Nevertheless, to indicate the best or most promising preservation methods considering multiple quality parameters is crucial to fill the gap in knowledge, guide further research on this subject, and thus commercially boost the application of UV-C light in fish and meat products.

In this context, this study aimed to identify the most promising preservation methods to potentialize the antimicrobial effect while minimizing the adverse effects of UV-C light on the oxidative stability and sensory attributes of fish and meat products through a systematic review.

## 2. Materials and Methods

This is a systematic review of the effects of UV-C light in combination with other preservation methods on fish and meat products, in which the articles were identified and screened according to the Preferred Reporting Items for Systematic Review and Meta-Analyses statement (PRISMA) [21]. Additionally, StArt^®^ software version 3.4 was used to aid in the exportation of the identified articles from the databases [22].

### 2.1. Searching Process

Aiming to identify the main databases and create the search strings from words and their synonyms used in the titles, abstracts, and keywords of articles concerning the subject of this study, an exploratory analysis was carried out on Google Scholar without establishing a time period or excluding patents and citations. Thereunto, search terms built according to the PICO (population = fish and meat products; intervention = preservation methods combined with UV-C light; comparison = treatment with no UV-C light; and outcome = safety and quality parameters during storage or not) in association with the Boolean operators were used as follows: (meat OR fish) AND combined AND UV-C AND (storage OR pathogen OR oxidation). After that, the search was performed in four reputable electronic databases in the Food Science and Technology field (Science Direct, PubMed, Web of Science, and Scopus) in the advanced search option on the title, abstract, and keywords and restricted to research articles published in the English language between 2000 and 2023 conforming to the following search strings and Boolean operators: (meat* OR fish* OR fillet* OR seafood) AND (combined* OR synergistic* OR hurdle*) AND (UV-C* OR ultraviolet) AND (“shelf life” OR “bacterial growth” OR “bacterial count” OR storage OR pathogen* OR decontamination OR inactivation OR “food safety” OR quality OR oxidation OR color OR texture OR sensory). Due to the limitation of Boolean connectors (maximum 8) in Science Direct, the search strings were adapted to this database as follows: (meat OR fish) AND (combined OR synergistic OR hurdle) AND (UV-C OR ultraviolet) AND (storage OR inactivation).

Subsequently, all identified articles were exported to the StArt^®^ software [22]. The entire database search process was carried out on 25 October 2022. It is worth highlighting that studies on the effect of UV-C in combination with other preservation methods on the quality parameters of fish and meat products stored under refrigeration are scarce. Therefore, an extensive search period was chosen to include as many studies on the subject as possible.

### 2.2. Screening and Eligibility Criteria 

The screening and eligibility processes of the identified articles were carried out in three stages. In the first step, duplicate articles were manually deleted. In the second step, each article’s title, abstract, and keywords were screened according to the eligibility criteria. All articles that met the inclusion criteria were accepted and selected to be read in full. The inclusion criteria were as follows: (I) studies evaluating pathogen reduction in fish and meat products using UV-C light in combination with other preservation methods; (II) studies concerning the combined effect of UV-C light with other preservation methods on shelf life (bacterial growth) of fish and meat products; (III) studies on the effect of UV-C light in combination with other preservation methods on oxidative degradation of fish and meat products, including lipid oxidation, protein oxidation, instrumental color and/or instrumental texture parameters; (IV) studies regarding the sensory evaluation of fish and meat products treated by combined UV-C light and other preservation methods.

**Figure 1:**
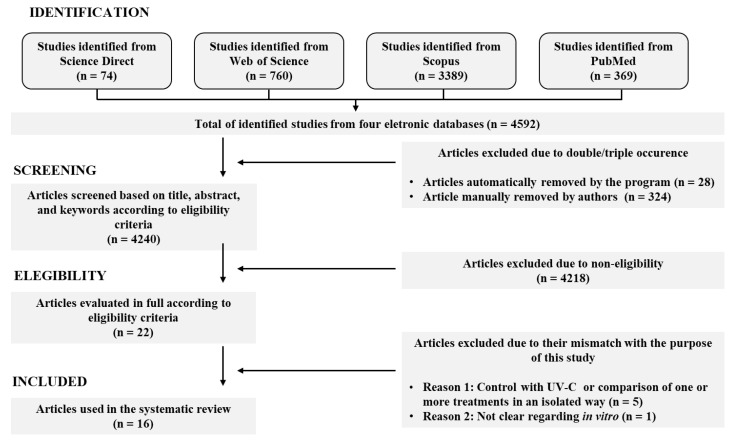
Results of the systematic literature search concerning the effects of UV-C light in combination with other preservation methods on fish and meat products between 2000 and 2023 through PRISMA flow diagram [21] and StArt^®^ software version 3.4 [22].

The established exclusion criteria were as follows: (I) studies on the combined effect of UV-C with other preservation methods in other food matrices instead of fish and meat products or other storage conditions instead of refrigeration; (II) studies without evaluation of UV-C light or with the evaluation of UV-C light in vitro/in vivo or other materials or with other UV types instead of UV-C; (III) studies without evaluation of the target parameters of this study in fish and meat products treated with combined UV-C light and other preservation methods; (IV) studies evaluating only the isolated effect of UV-C without combination with other preservation methods; (V) review papers; (VI) book chapters. All doubtful articles with ambiguous abstracts were not excluded; they were included in the third step, in which all selected articles were fully read, and those that did not meet the inclusion criteria were excluded (Figure 1). All database search processes, screening, and selection of eligible articles were carried out independently by two authors who later compared their results, resolving discrepancies by consensus.

### 2.3. Data Extraction

A total of 16 papers [5,20,23,24,25,26,27,28,29,30,31,32,33,34,35,36] were eligible for systematic review, and the following data were extracted: name of the first author followed by et al. (when applicable) and the year of publication of the study, specific food matrix (fish or meat product), control and combined UV-C treatments, average values of the parameters of interest (pathogen reduction, natural microbiota growth, thiobarbituric acid reactive substances (TBARS), carbonyl content, instrumental color and texture parameters, and data concerning sensory evaluation), temperature, and days of storage (when applicable). When needed, the UV-C doses were converted to J/cm^2^, and the parameters of interest were converted to a specific unit of measurement. All data were extracted separately for fish and meat products.

For data on pathogen reduction, microorganisms were grouped into Gram-positive and Gram-negative bacteria, and the percentage of reduction for each treatment compared to their control counterparts was calculated. For the evaluated parameters during a time period under refrigeration, the storage period and temperature were determined based on typical sampling days/temperature for each meat matrix. The storage period was 8 ± 2 days, and the storage temperature was 4 ± 1 °C.

Only the studies with fish species evaluated the natural microbiota growth during refrigerated storage, except for one evaluating ham [31]. Thus, we evaluated only the immediate effect of UV-C combined treatments on meat products. For fish, the mean values of the total aerobic psychrotrophic count (TAPC) were extracted from each day within the established storage period in this study (8 ± 2 days). After that, the primary predictive model of Baranyi and Roberts [37] (DMFit program; Institute of Food Research, Norwich, UK) was used to obtain the main microbial growth parameters as follows: lag phase and growth rate (µmax). The shelf life of each combined UV-C treatment and their control counterparts during refrigerated storage was established as the time needed for TAPC to reach 7 log CFU/g [38], considering the fitted values from DMFit.

The mean values of TBARS, carbonyl content, instrumental color, and texture parameters were extracted from each combined UV-C treatment and its control counterpart on the initial and final days of storage within the period established in this study (8 ± 2 days). Delta was calculated as the difference between the final day and the initial day, and subsequently, as the difference between the delta of each treatment and their corresponding control. Some TBARS results were expressed in absorbance for fish, making it impossible to convert them in mg malondialdehyde (MDA)/kg. Therefore, the delta percentage for each treatment in relation to the delta of its control counterpart was also calculated. For meat products, there have been no studies concerning storage. In this case, the delta was calculated as the difference between the mean values after treatments and their control counterparts within each parameter. 

The total color difference (∆E) was determined using the following equation [39]:

ΔE_final day-initial day_ = [(L* − L*)^2^ + (a* − a*)^2^ + (b* − b*)^2^]^1/2^ (fish) or

ΔE_treament-control_ = [(L* − L*)^2^ + (a* − a*)^2^ + (b* − b*)^2^]^1/2^ (meat products).

For fish, there was no comparison with the control because the main goal in calculating this parameter was to identify which combined UV-C treatments could maintain color changes imperceptible to consumers over refrigerated storage. 

Sensory data were described systematically because the eligible articles contained different methodologies for sensory assessment, making it impossible to compare them.

### 2.4. Risk of Bias 

The sources of bias in this study include some chosen specifications, such as the English language, search period (2000 to 2023), inclusion and exclusion criteria, and databases (Scopus, PubMed, Science Direct, and Web of Science). Moreover, other possible sources of bias may be the composition, biochemistry, and processing of each fish, and the composition, formulation, and technological processing of meat products.

## 3. Results and Discussion

### 3.1. Overview of Extracted Research

A total of 4592 studies were identified in the following online databases: Scopus (3389; 73.80%), Web of Science (760; 16.55%), PubMed (369; 8.04%), and Science Direct (74; 1.61%). Some studies (352) were deleted due to replicate conditions, and others (4218) were excluded from our inclusion criteria considering titles, abstracts, and keyword reading, leaving a total of 22 articles. These studies were fully read, and six of them were not within the eligibility criteria, resulting in 16 articles for this review (Figure 1).

### 3.2. Fish Species

#### 3.2.1. Immediate Effect on the Reduction of the Pathogenic Microbiota 

*Listeria monocytogenes*, *Listeria innocua*, *Staphylococcus aureus*, and *Bacillus cereus* were the Gram-positive pathogens evaluated in fish, while the Gram-negative ones were *Salmonella typhimurium*, *Salmonella enteritidis*, *Plesiomonas shigelloides*, *Aeromonas hydrophila*, and *Escherichia coli*, which are commonly involved in foodborne disease outbreaks [8].

Regarding the reduction of Gram-negative bacteria, the most effective UV-C combined treatment for reducing this pathogenic group was 0.5 J/cm^2^ + NTAP for 8 min (reduction of 33.83%; Table 1). Furthermore, several UV-C doses in combination with NTAP (0.02–0.5 J/cm^2^ for 1–8 min) and 2.4 J/cm^2^ + 35% ethanol reduced the Gram-negative bacteria by 18.74–24.06%, representing promising treatments. Otherwise, the least effective UV-C combined treatments were 0.6 J/cm^2^ + 35% ethanol, 0.6 or 1.2 J/cm^2^ + 70% ethanol, and modified atmosphere packaging (MAP; 50% CO_2_ and 50% N_2_) + 0.30 J/cm^2^, which reduced the Gram-negative bacteria by 1.44–7.11%; Table 1). 

Concerning the reduction of Gram-positive pathogens, the best UV-C combined treatment was 1% Verdad N6 + 0.05 J/cm^2^ + VP, which reduced it by 25.81% (Table 1). As for the reduction of Gram-negative bacteria, several UV-C doses combined with NTAP (0.05–0.5 for 1–8 min) and ethanol (2.4 J/cm^2^ + 35 or 70%) demonstrated a promising reduction of Gram-positive bacteria (18.00–21.88%). On the other hand, the worst UV-C combined treatments for reducing Gram-positive bacteria in fish species were 0.02 or 0.05 J/cm^2^ + NTAP for 2 min, 0.02 J/cm^2^ + NTAP for 1 min, 3.09 J/cm^2^ + acidified electrolyzed water (AEW) for 1 min, 1.2 J/cm^2^ + 70% ethanol, and 0.6 J/cm^2^ + 35% ethanol, which reduced this pathogenic group by 7.77–11.53% (Table 1).

NTAP may enhance the UV-C penetration power through the ionization of gases generating free radicals, reactive nitrogen species (RNS), and ROS, in addition to the emission of photons, which synergistically leads to integrity loss of microbial DNA [40,41]. Nevertheless, these authors stated that the effect of NTAP depends on the treatment conditions (e.g., voltage, gas type, mode of plasma exposure, and treatment time) and food matrix. In our study, the best conditions in combination with UV-C light for reducing Gram-negative pathogens in fish were direct exposure to nitrogen at atmospheric pressure and a voltage of 1 kHz for 8 min. Otherwise, 1% of Verdad N6 in vacuum-packed fish was the most efficient preservation method combined with UV-C to reduce Gram-positive pathogens. Verdad N6 is a commercially available white distilled vinegar that can acidify the interior of pathogens through the penetration of undissociated acetic acid into the bacterial membrane [24]. 

#### 3.2.2. Effect on Overall Fish Quality during Storage at 4 ± 1 °C for 8 ± 2 Days

##### Microbial Parameters and Shelf-Life

Based on the total aerobic psychrotrophic count (TAPC), MAP (80% CO_2_:20% N_2_) + 0.1 J/cm^2^ increased the lag phase in 4.68 days and reduced the µmax by 0.22 log CFU/g/h throughout the refrigerated storage, resulting in a shelf-life extension by at least four days. MAP (50% CO_2_:50% N_2_) + 0.30 J/cm^2^ also positively affected both the lag phase (an increase of 1.75 days) and µmax (decrease of 0.26 log CFU/g/h) during over-refrigerated storage, increasing the shelf life by at least 5 days. UV-C at 0.102 J/cm^2^ + Ageless SS-50 increased the lag phase by 3.01 days; however, it also increased the µmax by 0.50 log CFU/g/h, prolonging the shelf life by at least 2 days. This shelf-life extension was also observed for VP + 0.1 J/cm^2^, VP + 0.30 J/cm^2^, and 0.301 J/cm^2^ + Ageless SS-50. UV-C at 0.103 J/cm^2^ + HHP (220 MPa/10 min) decreased the lag phase by 0.54 days and the µmax by 1.47 log CFU/g/h, extending the shelf life by at least 2 days under aerobic conditions (Table 2). 

All eligible studies evaluated freshwater fish species during storage under refrigeration. The microbiota of this fish group is mainly composed of Gram-negative aerobic and facultative anaerobic bacteria. Nevertheless, *Pseudomonas* spp. is the predominant group with an increase in the refrigerated storage period [42]. As *Pseudomonas* spp. are Gram-negative obligate aerobic bacteria, they are extremely sensitive to carbon dioxide and low oxygen concentration, as well as bacteria from the Enterobacteriaceae family, which are Gram-negative facultative anaerobic bacteria [43]. Our results may be explained by the absence of oxygen and the mixture of gases from modified atmosphere packaging, wherein nitrogen (N_2_) is an inert gas and carbon dioxide (CO_2_) is responsible for the bacteriostatic effect through penetration into microbial membranes, changing cell membrane function and protein properties [25,44,45]. The oxygen absorber combined with UV-C also showed promising results since it removed O_2_ and increased the CO_2_ level inside the packaging [46].

##### Lipid Oxidation

Five UV-C treatments combined with MAP or NTAP under vacuum storage strongly increased the lipid oxidation (LOX) in fish species stored under refrigeration for 8 ± 2 days as follows: MAP (50% CO_2_ and 50% N_2_) + 0.30 J/cm^2^ (1128.57%), 0.5 J/cm^2^ + NTAP for 4 min (577.78%), 0.1 or 0.05 J/cm^2^ + NTAP for 4 min, 0.1 J/cm^2^ + NTAP for 2 min (140.74−281.48%), 0.5 J/cm^2^ + NTAP for 1 min, 2.4 J/cm^2^ + 35% ethanol, and 0.02 J/cm^2^ + NTAP for 4 min (20–62.96%; Table 2). On the other hand, other UV-C combined treatments decreased the LOX of fish species throughout the refrigerated storage by 6.67–10.00% (MAP at 80% CO_2_:20% N_2_ + 0.1 J/cm^2^ and 0.103 J/cm^2^ + HHP at 220 MPa/10 min; VP + 0.30 J/cm^2^) and by 25.17–33.33% (VP + 0.1 J/cm^2^, and 0.102 or 0.301 J/cm^2^ + Ageless SS-50). The best ones for LOX were 0.103 J/cm^2^ + HHP at 220 MPa/10 min, 2.4 J/cm^2^ + 70% ethanol, and 0.102 or 0.301 J/cm^2^ + Ageless SS-50, thus decreasing this parameter by 64.52–80% (Table 2). 

The indirect action of UV-C is based on ROS generation, which commonly leads to enhanced lipid oxidation [11]. Our findings indicated that HHP at 220 MPa/10 min, 70% ethanol, and an oxygen absorber (Ageless SS-50) were the most effective in minimizing lipid oxidation induced by UV-C (0.1–0.3 J/cm^2^). HHP may prevent lipid oxidation by lipoxygenase inactivation depending on the treatment conditions [47], whereas the oxygen absorber sachet oxidizes iron spontaneously, reducing and maintaining O_2_ levels to less than 0.01% inside the package [46,48,49]. In general, lipid oxidation in fish is enhanced by HHP above 300 MPa [50]. Ethanol is a widely used sanitizer due to its low cost and effectiveness in inhibiting the growth of microorganisms [51], contributing to the higher pH stability of fish during storage, which is close to neutrality. According to Mozuraityte et al. [52], lipid oxidation is less intense at a pH of about 7, considering a range from 3.3 to 7.0. 

It is worth highlighting that the evolution of the oxidative process depends on the balance between the antioxidant and pro-oxidative systems related to the intrinsic particularities of each fish species (e.g., enzymatic activities and protein and lipid compositions) and extrinsic factors, such as packaging conditions [29,30]. In our study, the treatment with 0.103 J/cm^2^ + HHP (220 MPa/10 min) was better in tilapia fillets stored in a vacuum (LOX reduced by 80%) than in air-packaging (LOX reduced by 8.85%), obviously due to low O_2_ level in vacuum systems. Moreover, 0.102 or 0.301 J/cm^2^ + Ageless SS-50 was assessed in tilapia and trout fillets, which reduced LOX by about 65% and 25%, respectively, regardless of the UV-C dose. This may be explained by the trout having predominantly dark muscles, whereas tilapia have light ones. Dark flesh contains more myoglobin, proteases, lipids, and unsaturated to saturated fatty acids ratio, which favors oxidative degradation [53]. It is also worth highlighting that 2.4 J/cm^2^ + 70% ethanol was highly effective against lipid oxidation since it was applied in an air-packed dark flesh fish treated with a high UV-C dose. On the other hand, MAP (50% CO_2_ and 50% N_2_) + 0.30 J/cm^2^ was the worst combined treatment increasing lipid oxidation. This may be attributed to the ability of CO_2_ to denature proteins and release iron, which catalyzes lipid oxidation [54].

##### Protein Oxidation

Protein oxidation occurs similarly to lipid oxidation, generating a complex free radical chain reaction by removing one hydrogen atom, mainly of functional groups, from the side chains of amino acid, resulting in adverse changes in color and texture [48,53].

Only three studies have evaluated the effect of UV-C combined treatments on the carbonyl content of fish species stored under refrigeration (Table 2). Monteiro et al. [29] observed that the oxygen absorber (Ageless SS-50) in combination with two UV-C doses (0.102 or 0.301 J/cm^2^) reduced the protein oxidation of tilapia fillets by about 2.6 nmol carbonyl/mg protein (49%) during storage under refrigeration for 8 ± 2 days. Otherwise, 0.102 J/cm^2^ + Ageless SS-50 and 0.301 J/cm^2^ + Ageless SS-50 decreased the carbonyl content of rainbow trout fillets by 1.89 and 0.91 nmol carbonyl/mg protein (61.17 and 29.45%), respectively, over the refrigerated storage [30]. The lower effectiveness of the same combined treatments in trout than in tilapia fillets may be attributed to differences in muscle composition and biochemistry between the two fish species, as mentioned above. Another interesting finding was that the Ageless SS-50 reduced lipid oxidation equally in both tilapia and trout regardless of UV-C dose, while it doubled the reduction of protein oxidation in trout only when combined with the low UV-C dose (0.102 J/cm^2^).

The treatment 0.103 J/cm^2^ + HHP (220 MPa/10 min) increased the carbonyl content of tilapia fillets by 0.18 nmol carbonyl/mg protein (20%) during storage under aerobic conditions at 4 ± 1 °C for 8 ± 2 days [28]. On the other hand, this combined treatment decreased the lipid oxidation of air-packed tilapia fillets during refrigerated storage. These facts may suggest that proteins are more sensitive to UV-C and HHP than lipids. 

##### Instrumental Color Parameters

During refrigerated storage, protein denaturation naturally occurs, leading to exposure of hydrophobic groups, which reduces water retention and changes the meat’s reflectance by increasing L* values [28]. Moreover, a* and b* values usually change during the refrigerated storage of fish species. Meat discoloration is associated with a* changes due to the auto-oxidation of myoglobin that occurs when oxymyoglobin is transformed into metmyoglobin by converting ferrous iron (Fe^2+^) to ferric iron (Fe^3+^) [55,56]. Discoloration is generally indicated by reduced a* values in dark-muscle fish and increased a* values in white-muscle fish [29,30]. On the other hand, the increase in b* values may be related to increased lipid oxidation in refrigerated fish species throughout the storage period [29]. 

Among the UV-C combined treatments evaluated in white fish species, 0.103 J/cm^2^ + HHP (220 MPa/10 min) increased L* and b* values by 14.54 and 7.97, respectively, while slightly decreasing a* value by 0.25 during refrigerated storage under aerobic conditions for 8 ± 2 days (Table 2). MAP (50% CO_2_:50% N_2_) + 0.30 J/cm^2^ and VP + 0.30 J/cm^2^ increased L*, a*, and b* values by 0. 42–0.49, 2.54–2.15, and 1.31–0.18, respectively, during the entire refrigerated storage period. On the other hand, 0.102 or 0.301 J/cm^2^ + oxygen absorber (Ageless SS-50) decreased L* (1.65–3.84), a* (0.54–0.57), and b* (33.53–34.72) values during the storage under refrigeration. In terms of UV-C combined treatments in dark fish species, those with oxygen absorber (0.102 or 0.301 J/cm^2^ + Ageless SS-50) and ethanol under aerobic storage (2.4 J/cm^2^ + 35% or 70% ethanol) preserved more a* values (0.20–0.82) throughout the refrigerated storage, mainly due to the treatments combined with oxygen absorbers. Furthermore, 0.102 or 0.301 J/cm^2^ + Ageless SS-50 decreased L* (0.79–1.17) and b* values (1.62–1.65), whereas 2.4 J/cm^2^ + 35% or 70% ethanol increased L* values by 0.43–0.50, and decreased (2.4 J/cm^2^ + 35% ethanol) or increased (2.4 J/cm^2^ + 70% ethanol) b* values. The treatments 0.05 J/cm^2^ + NTAP for 4 min, 0.1 J/cm^2^ + NTAP for 2 min, and 0.5 J/cm^2^ + NTAP for 1 min decreased L* (1.80–2.64), a* (1.73–2.47), and b* (1.13–4.76) values of vacuum-packed dark fish species stored under refrigeration for 8 ± 2 days (Table 2).

Regarding total color difference (ΔE), it sums up the overall modifications of L*, a*, and b* values, inferring a perceptible color change by consumers if ΔE is equal to or above 5 [57]. For white fish species, the worst treatment was 0.103 J/cm^2^ + HHP (220 MPa/10 min) evaluated under aerobic conditions (ΔE = 17.73), while the best treatments for preserving color changes were 0.301 J/cm^2^ + Ageless SS-50 (ΔE = 2.89) and 0.102 J/cm^2^ + Ageless SS-50 (ΔE = 4.51). VP + 0.30 J/cm^2^ and MAP (50% CO_2_:50% N_2_) + 0.30 J/cm^2^ showed ΔE higher than 5 (ΔE = 5.67 and 6.28, respectively). 

HHP (150–300 MPa) is well known for increasing the total color difference in fish, resulting in cooked meat appearance, which has been associated mainly with protein denaturation, lipid and protein oxidation, and changes in muscle hydration properties [58,59], and may have been accelerated by aerobic storage conditions. Considering the same UV-C dose (0.3 J/cm^2^) combined with VP, MAP, and O_2_ absorbers, the latter was the only one leading to non-perceptible changes in human eyes. The O_2_ scavenger reduces and maintains the O_2_ concentration inside the package at levels less than 0.01%, whereas in the VP and MAP systems, O_2_ can penetrate through the packaging over the storage period [32,46,48]. Furthermore, the low O_2_ levels in the VP and MAP may have been overcome by the high UV-C dose. MAP still resulted in more color changes than VP, possibly due to the CO_2_ denatures protein favoring myoglobin oxidation and reducing the pH on the surface (CO_2_ dissolution), decreasing the protein water-holding capacity, and increasing the drip [54].

For dark fish species, the treatments showing ΔE lower than 5 were 2.4 J/cm^2^ + 35% (ΔE = 1.21) or 70% ethanol (ΔE = 1.46) under aerobic storage. UV-C at 0.102 J/cm^2^ + Ageless SS-50 demonstrated ΔE close to 5, indicating a potential result (ΔE = 5.85). Although some treatments have decreased a* values, they also decreased L* and b* values during the entire refrigerated storage under vacuum conditions, resulting in the following promising results concerning total color difference: 0.05 J/cm^2^ + NTAP for 4 min (ΔE = 2.64), 0.5 J/cm^2^ + NTAP for 1 min (ΔE = 2.80), and 0.1 J/cm^2^ + NTAP for 2 min (ΔE = 5.19). The successful results for ethanol preventing discoloration may be attributed to its great capacity to stabilize fish pH even under high UV-C doses and aerobic storage conditions. According to some authors, oxymyoglobin and unsaturated fatty acids are more stable in neutral environments [52,60], and fish have a pH close to neutrality. The O_2_ absorbers were also effective in dark-muscle fish due to their mechanism of action previously mentioned. Regarding the combined NTAP with UV-C, as their mechanisms of action lead to the generation of free radicals, they induce oxidation and color changes in a dependent manner (time and UV-C dose). Therefore, some combined conditions may result in no or minor color modifications that are not perceptible to consumers [23]. Vacuum storage can help to achieve this great result. 

##### Instrumental Texture Parameters

During refrigerated storage of fish species, hardness and chewiness decrease due to proteolysis by the action of endogenous and microbial proteases [61]. 

The results of hardness (HA), chewiness (CW), cohesiveness (CO), and springiness (SP) are shown in Table 2. CO and SP were unaffected by all UV-C combined treatments.

The worst treatment for the texture of fish species during refrigerated storage was 0.103 J/cm^2^ + HHP (220 MPa/10 min) under aerobic conditions by decreasing HA and CW by 7.72 N (41.13%) and 1.51 N × mm (54.91%), respectively. The other UV-C combined treatments increased HA and CW. The treatments MAP (50% CO_2_:50% N_2_) + 0.30 J/cm^2^ and VP + 0.30 J/cm^2^ increased HA in 7.04 (87.34%) and 0.52 N (6.45%) and CW in 1.79 (33.84%) and 2.33 N × mm (44.05%), respectively. The UV-C treatments combined with an oxygen absorber (Ageless SS-50) applied in both white and dark fish species increased HA by 1.30–3.45 N (6.99–18.61%) and CW by 0.50–0.73 N × mm (5.87–8.61%) throughout the refrigerated storage. 

The effect of UV-C on the texture parameters was highly variable. It may decrease the availability of microbial proteases by reducing the microbial growth rate, thereby preventing fish softness, but it may also favor proteolysis by inducing myofibrillar protein denaturation, thereby softening the fish more [11,62]. The results for UV-C + HHP found in this study can be explained by an increase in the susceptibility of proteins to high-pressure effects due to UV-C-induced protein denaturation [28]. On the other hand, UV-C combined with treatments containing none or low O_2_ levels (MAP, VP, and O_2_ absorber) resulted in better texture results, mainly with MAP at 50% CO_2_:50% N_2_, due to the less oxidative environment preventing protein breakdown associated with their antimicrobial effect, corroborating with our findings for microbial parameters and shelf life. 

##### Sensory Quality 

Only two studies have evaluated sensory attributes in which fish were treated with UV-C combined with NTAP [23], UV-C in combination with ultrasound (US), and UV-C combined with US and AEW [27]. Colejo et al. [23] investigated the treatments through the duo-trio test using 32−48 semi-trained panelists. These authors reported that UV-C at 0.05 J/cm^2^ for 1 min and UV-C at 0.1 J/cm^2^ for 2 min did not change the fish appearance until days 14 and 7 of refrigerated storage, respectively; however, UV-C at 0.5 J/cm^2^ for 4 min led to appearance changes on 7th day of storage. Mikš-Krajnik et al. [27] evaluated odor, color, and texture using 36 untrained panelists through a scoring system of 3 points (3 = very good, 2 = good, and 1 = unacceptable). These authors observed that the treatments did not affect the texture, the odor was equally decreased by UV-C (3.09 J/cm^2^) + US (45 kHz, 200 W for 1 min) and UV-C (3.09 J/cm^2^) + US (45 kHz, 200 W for 1 min) + AEW (1 min), and color was more changed by triple combination than the double one. Despite these facts, Mikš-Krajnik et al. [27] reported that no treatment was considered unacceptable. 

### 3.3. Meat Products

#### 3.3.1. Immediate Effect on the Reduction of the Pathogenic Microbiota 

The Gram-negative pathogens evaluated in meat products were *Escherichia coli* O157:H7, *Escherichia coli* K-12, *Yersinia enterocolitica*, and *S. typhimurium*, whereas the Gram-positive ones were *L. monocytogenes* and *L. innocua*. The meat products investigated were ham, sliced Bologna meat, sliced Brazilian dry-cured loins, and frankfurters. 

For the Gram-negative bacteria, the best treatments were UV-C at 0.11 and 0.13 J/cm^2^ combined with nir-infrared (NIR) heating (NIR-H; 200.36 µW/cm^2^/nm), reducing this pathogen group in 52.09% and 70.82%, respectively. Other treatments reduced the Gram-negative bacteria by 18.38–28.36%, 32.36–43.36%, and 6.40–16.00% (Table 3).

The Gram-positive bacteria were more effectively reduced (58.89–67.77%) by 0.13 J/cm^2^ combined with NIR-H (200.36 µW/cm^2^/nm), UV-C at 1, 2, or 4 J/cm^2^ with flash pasteurization (FP) during 1.5 or 3 s, and UV-C at 2 J/cm^2^ with FP for 0.75 s. Lower reduction ranges were observed with other UV-C combined treatments, such as 24.91–35.64%, 43.50–49.64%, and 55.57–56.10% (Table 3). 

Infrared (IR) radiation promotes heating through electromagnetic waves with no influence on the air surrounding food [63,64], and has demonstrated promising findings when applied simultaneously with UV-C light since it increases the penetration capacity of the photons from UV using less energy, which preserves the original meat quality [20]. Regarding synergistic antibacterial effects, Ha and Kang [20] reported that simultaneous NIR-UV treatment damages cell envelopes and ribosomes, inhibiting protein synthesis and the capacity for repairing injured cells.

Flash pasteurization (FP) is a short-steam propulsion system applied to sausage emulsions [65]. UV-C causes DNA breakage, hindering its replication and making bacterial cells more susceptible to thermal treatments, which can explain the effectiveness of the combination of UV-C and FP [34].

#### 3.3.2. Instrumental Color Parameters

Overall, UV-C combined treatments reduced the L*, a*, and b* values (Table 4). The best UV-C combined treatments concerning total color difference were in descending order as follows: 0.4 J/cm^2^ + MAP (70% O_2_ and 30% N_2_) (ΔE = 2.26), 5% lauric-arginate ester (LAE) + 0.5 J/cm^2^ and 11% lactic acid (LA) + 0.55 J/cm^2^ (ΔE = 2.57), 6.5% LA + 0.01 J/cm^2^ (ΔE = 2.91), 4.08 J/cm^2^ + MAP (70% O_2_ and 30% N_2_; ΔE = 3.28), 6.5% LA + 0.64 J/cm^2^ (ΔE = 3.70), 2% LA + 0.55 J/cm^2^ (ΔE = 3.86), 2% LA + 0.1 J/cm^2^ (ΔE = 3.97), 0.1% LA + 0.33 J/cm^2^ (ΔE = 4.27), and 12.9% LA + 0.33 J/cm^2^ (ΔE = 4.44) (Table 4). The color differences in cured meat products are driven mainly by changes in redness. Although a drop in pH leads to protein denaturation and, thereby, oxidative processes [33], UV-C under vacuum may form hydroxyl radical, which recombines with other produced substances, resulting in the formation of H_2_O_2_. This compound combined with Fe^3+^ leads to Fe^2+^ reduction (Fenton-like reaction) [66,67], preserving the decrease in the a* values. The effectiveness of MAP (70% O_2_ and 30% N_2_) may be explained by the absence of CO_2_ (less acidification) and the low amount of fat in the meat product (ham; 2% fat), resulting in a low rate of lipid oxidation. According to Wang et al. [68], MDA interacts directly with myoglobin amino acid residues, increasing its susceptibility to oxidation by exposing it to a highly oxidative medium. 

#### 3.3.3. Lipid and Protein Oxidation

Only one study evaluated the MDA and carbonyl levels of meat products (sliced Brazilian dry-cured loin) treated with LA (0.1–12.9%), VP, and UV-C (0.01–0.64 J/cm^2^) [33]. All the combined UV-C treatments increased lipid (100.00–258.06%) and protein oxidation (5.58–52.79%; Table 4). The authors found that the greater the UV-C dose, the greater the lipid oxidation, but the increased LA addition tended to reduce this parameter, which was attributed to the proximity of the pH to the isoelectric point, decreasing the lipid oxidation catalyzed by iron [69]. Concerning protein oxidation, it was affected by the interaction between LA and UV-C, but their synergistic mechanisms are still unknown. The best combinations were 2% LA + 0.1 J/cm^2^ for lipid oxidation and 7.7% LA + 0.36 J/cm^2^ for protein oxidation, whereas the worst ones were 6.5% LA + 0.64 J/cm^2^ and 11% LA + 0.55 J/cm^2^, respectively. Among the promising combined LA and UV-C treatments for preserving the color, 2% LA + 0.1 J/cm^2^ was the least increased lipid oxidation (100%), and 2% LA + 0.55 J/cm^2^ was the least enhanced protein oxidation (14.16%). 

#### 3.3.4. Instrumental Texture Parameters

Two studies have investigated the effect of UV-C combined treatments with FP or LAE solution on the shear force of vacuum-packed frankfurters [34,35], and none significantly affected the texture (UV-C at 1 J/cm^2^ + FP for 1.5 s, 2 J/cm^2^ + FP for 3 s, and 5% LAE + 0.5 J/cm^2^; Table 4).

## 4. Conclusions and Future Perspectives

Based on our findings, combining UV-C with other technologies could be a promising cost-effective way to enable their industrial application in fish and meat products. Nevertheless, the best UV-C combined treatments have not been evaluated for overall quality parameters, including sensorially, indicating a gap in the literature. Therefore, further optimization studies considering the effect of these promising UV-C combined treatments on the overall quality (pathogen reduction, shelf life, physicochemical parameters, and sensory quality) of fish and meat products are needed. Moreover, MAP (50% CO_2_:50% N_2_) + 0.30 J/cm^2^ should be evaluated in combination with antioxidant technologies because this combined treatment was not only the best for prolonging fish shelf life (at least 5 days) but also for enhancing oxidative degradation. Other UV sources, such as excilamps with a gas mixture (KrCl) through dielectric barrier discharge emitting radiation at 222 nm, have shown promising bacterial inactivation findings and should be further studied in fish and meat products. 

## Figures and Tables

**Table 1:** Reduction of Gram-negative and Gram-positive bacteria by UV-C combined treatments in fish.

Gram-Negative Bacteria	Treatments *	Reduction (%)	Reference
*S. typhimurium*, *S. enteritidis*, *P. shigelloides*, *A. hydrophila*, and *E. coli*	0.02 J/cm^2^ + NTAP 1 min	10.91	Colejo et al. (2018) [23]
	0.05 J/cm^2^ + NTAP 1 min	12.74	
	0.1 J/cm^2^ + NTAP 1 min	16.74	
	0.5 J/cm^2^ + NTAP 1 min	19.43	
	0.02 J/cm^2^ + NTAP 2 min	14.74	
	0.05 J/cm^2^ + NTAP 2 min	16.86	
	0.1 J/cm^2^ + NTAP 2 min	18.74	
	0.5 J/cm^2^ + NTAP 2 min	21.49	
	0.02 J/cm^2^ + NTAP 4 min	15.71	
	0.05 J/cm^2^ + NTAP 4 min	18.80	
	0.1 J/cm^2^ + NTAP 4 min	20.46	
	0.5 J/cm^2^ + NTAP 4 min	22.97	
	0.02 J/cm^2^ + NTAP 8 min	20.11	
	0.05 J/cm^2^ + NTAP 8 min	22.91	
	0.1 J/cm^2^ + NTAP 8 min	24.06	
	0.5 J/cm^2^ + NTAP 8 min	33.83	
*S. typhimurium* and *E. coli* O157:H7	VP + 0.30 J/cm^2^	15.28	Lázaro et al. (2020) [25]
	MAP (50% CO_2_ and 50% N_2_) + 0.30 J/cm^2^	5.51	
*E. coli*	0.6 J/cm^2^ + 35% ethanol	1.44	Lee et al. (2019) [26]
	1.2 J/cm^2^ + 35% ethanol	13.56	
	2.4 J/cm^2^ + 35% ethanol	22.33	
	0.6 J/cm^2^ + 70% ethanol	5.25	
	1.2 J/cm^2^ + 70% ethanol	7.11	
	2.4 J/cm^2^ + 70% ethanol	17.26	
**Gram-Positive Bacteria**	**Treatments**	**Reduction (%)**	**Reference**
*L. monocytogenes*, *L. innocua*, and *S. aureus*	0.02 J/cm^2^ + NTAP 1 min	7.81	Colejo et al. (2018) [23]
	0.05 J/cm^2^ + NTAP 1 min	11.52	
	0.1 J/cm^2^ + NTAP 1 min	14.48	
	0.5 J/cm^2^ + NTAP 1 min	18.00	
	0.02 J/cm^2^ + NTAP 2 min	10.48	
	0.05 J/cm^2^ + NTAP 2 min	13.71	
	0.1 J/cm^2^ + NTAP 2 min	17.05	
	0.5 J/cm^2^ + NTAP 2 min	19.52	
	0.02 J/cm^2^ + NTAP 4 min	12.86	
	0.05 J/cm^2^ + NTAP 4 min	16.38	
	0.1 J/cm^2^ + NTAP 4 min	18.38	
	0.5 J/cm^2^ + NTAP 4 min	20.38	
	0.02 J/cm^2^ + NTAP 8 min	16.19	
	0.05 J/cm^2^ + NTAP 8 min	18.48	
	0.1 J/cm^2^ + NTAP 8 min	21.05	
	0.5 J/cm^2^ + NTAP 8 min	21.81	
*B. cereus*	0.6 J/cm^2^ + 35% ethanol	7.77	Lee et al. (2019) [26]
	1.2 J/cm^2^ + 35% ethanol	15.54	
	2.4 J/cm^2^ + 35% ethanol	21.88	
	0.6 J/cm^2^ + 70% ethanol	0.89	
	1.2 J/cm^2^ + 70% ethanol	11.53	
	2.4 J/cm^2^ + 70% ethanol	19.29	
*L. monocytogenes*	3.09 J/cm^2^ + AEW	11.40	Mikš-Krajnik et al. (2017) [27]
	3.09 J/cm^2^ + US	15.80	
	3.09 J/cm^2^ + US + AEW	15.00	
*L. monocytogenes*	1% Verdad N6 + VP + 0.050 J/cm^2^	25.81	Heir et al. (2019) [24]

* NTAP—non-thermal atmospheric plasma; MAP—modified atmosphere packaging; VP—vacuum packaging; US—ultrasound; and AEW—acidified electrolyzed water.

**Table 2:** Bacteriological (lag phase and µmax based on total aerobic psychrotrophic count—TAPC) and physicochemical (lipid oxidation, lightness, redness, yellowness, total color difference, hardness, chewiness, cohesiveness, and springiness) parameters of fish subjected to UV-C combined treatments stored at 4 ± 1 °C for 8 ± 2 days.

Treatments *	Lag Phase	µmax	LOX	PROTOX	L*/a*/b*/∆E	HA/CW/CO/SP	Reference
0.02 J/cm^2^ + NTAP 4 min + VP	NA ^£^	NA	↑25.93	NA	NA	NA	Colejo et al. (2018) [23]
0.05 J/cm^2^ + NTAP 4 min + VP	NA	NA	↑188.89	NA	↓1.91/↓2.04/↓4.76/2.64	NA	
0.1 J/cm^2^ + NTAP 2 min + VP	NA	NA	↑140.74	NA	↓2.64/↓2.47/↓1.13/5.19	NA	
0.1 J/cm^2^ + NTAP 4 min + VP	NA	NA	↑281.48	NA	NA	NA	
0.5 J/cm^2^ + NTAP 1 min + VP	NA	NA	↑62.96	NA	↓1.80/↓1.73/↓1.17/2.80	NA	
0.5 J/cm^2^ + NTAP 4 min + VP	NA	NA	↑577.78	NA	NA	NA	
0.103 J/cm^2^ + HHP (220 MPa/10 min) ^₭^	↓0.54	↓1.47	↓80.00	NA	NA	NA	Monteiro et al. (2018) [5]
0.102 J/cm^2^ + Ageless SS-50 ^€^	↑3.01	↑0.50	↓65.59	↓2.57	↓1.65/↓0.57/↓1.17/4.51	↑3.45/↑0.50/0.014/0.007	Monteiro et al. (2020) [29]
0.301 J/cm^2^ + Ageless SS-50 ^€^	↑1.01	↑0.02	↓64.52	↓2.58	↓3.84/↓0.54/↓1.13/2.89	↑1.30/↑0.73/0.005/0.007	
VP + 0.30 J/cm^2^	↑0.70	↓0.13	↓10.00	NA	↑0.49/↑2.15/↑0.18/5.67	↑0.52/↑2.33/0.022/0.015	Lázaro et al. (2020) [25]
MAP (50% CO_2_ and 50% N_2_) + 0.30 J/cm^2^	↑1.75	↓0.26	↑1128.57	NA	↑0.42/↑2.54/↑1.31/6.28	↑7.04/↑1.79/0.042/0.065	
2.4 J/cm^2^ + 35% ethanol	NA	NA	↑20.00	NA	↑0.43/↑0.67/↓0.37/1.21	NA	Lee et al. (2019) [26]
2.4 J/cm^2^ + 70% ethanol	NA	NA	↓80.00	NA	↑0.50/↑0.20/↑0.50/1.46	NA	
0.103 J/cm^2^ + HHP (220 MPa/10 min) ^₩^	NA	NA	↓8.85	↑0.18	↑14.54/↓0.25/↑7.97/17.73	↓7.72/↓1.51/0.030/0.010	Monteiro et al. (2019) [28]
0.102 J/cm^2^ + Ageless SS-50 ^¥^	NA	NA	↓27.12	↑1.20	↓1.17/↑0.82/↓1.62/5.85	↑2.82/↑0.57/0.018/0.001	Monteiro et al. (2020) [30]
0.301 J/cm^2^ + Ageless SS-50 ^¥^	NA	NA	↓25.17	↑2.18	↓0.79/↑0.82/↓1.65/6.22	↑2.74/↑0.51/0.002/0.011	
VP + 0.1 J/cm^2^	↑0.52	↓0.06	↓33.33	NA	NA	NA	Rodrigues et al. (2016) [32]
MAP (80% CO_2_ and 20% N_2_) + 0.1 J/cm^2^	↑4.68	↓0.22	↓6.67	NA	NA	NA	

Values are means obtained from the difference between the final day and initial day of storage and further compared to their control counterparts, which is indicated by symbols ↑ or ↓ (increase or decrease in relation to control over refrigerated storage), except for the total color difference (∆E). In this case, values indicate color changes in each specific treatment without comparison with the control. For instrumental texture parameters, values without symbols indicate that the difference from their control counterparts was insignificant. ^£^ NA—not analyzed; * MAP—modified atmosphere packaging; NTAP—non-thermal atmospheric plasma; VP—vacuum packaging; HHP—high hydrostatic pressure. Lag phase in days; µmax (exponential growth rate) in log CFU (colony-forming unit)/g/h; LOX (lipid oxidation) in %; PROTOX (protein oxidation) in nmol carbonyls/mg protein; L*—lightness; a*—redness; b*—yellowness; ∆E—total color difference; HA (hardness) in Newton (N); CW (chewiness) in N × mm; CO (cohesiveness) in ratio; SP (springiness) in ratio. ^€^ Combined treatments applied in tilapia fillets. ^¥^ Combined treatments applied in rainbow trout fillets. ^₭^ Fish stored in vacuum packaging during refrigerated storage. ^₩^ Fish stored in air-packaging during refrigerated storage.

**Table 3:** Reduction of Gram-negative and Gram-positive bacteria with UV-C combined treatments in meat products.

Gram-Negative Bacteria	Treatments *	Reduction (%)	Reference
*E. coli* O157:H7 and *S. typhimurium*	0.02 J/cm^2^ + NIR-H	28.36	Ha and Kang (2015) [20]
	0.04 J/cm^2^ + NIR-H	32.36	
	0.055 J/cm^2^ + NIR-H	33.73	
	0.07 J/cm^2^ + NIR-H	37.27	
	0.09 J/cm^2^ + NIR-H	43.36	
	0.11 J/cm^2^ + NIR-H	52.09	
	0.13 J/cm^2^ + NIR-H	70.82	
*S. typhimurium*	0.01 J/cm^2^ + 6.5% LA	13.24	Rosário et al. (2021) [33]
	0.1 J/cm^2^ + 2% LA	14.71	
	0.1 J/cm^2^ + 11% LA	19.12	
	0.55 J/cm^2^ + 2% LA	23.53	
	0.55 J/cm^2^ + 11% LA	11.76	
	0.64 J/cm^2^ + 6.5% LA	25.00	
	0.33 J/cm^2^ + 0.1% LA	14.71	
	0.33 J/cm^2^ + 12.9% LA	20.59	
	0.33 J/cm^2^ + 6.5% LA	18.38	
	0.36 J/cm^2^ + 7.7% LA	19.12	
*S. enteriditis*, *S. typhimurium*, and *Salmonella* Senftenberg	0.5 J/cm^2^ + 5% LAE + VP	38.67	Sommers et al. (2010) [35]
*E. coli* K-12	VP-PE + 0.045 J/cm^2^	10.40	Tarek et al. (2015) [36]
	VP-PE + 0.070 J/cm^2^	12.13	
	VP-PE + 0.138 J/cm^2^	15.47	
	VP-PE + 0.270 J/cm^2^	18.67	
	VP-PE + 0.405 J/cm^2^	18.93	
	VP-OPP + 0.021 J/cm^2^	7.33	
	VP-OPP + 0.051 J/cm^2^	9.07	
	VP-OPP + 0.101 J/cm^2^	9.47	
	VP-OPP + 0.207 J/cm^2^	14.27	
	VP-OPP + 0.305 J/cm^2^	16.00	
	VP-ClearTite + 0.018 J/cm^2^	7.20	
	VP-ClearTite + 0.024 J/cm^2^	6.40	
	VP-ClearTite + 0.059 J/cm^2^	7.33	
	VP-ClearTite + 0.127 J/cm^2^	10.27	
	VP-ClearTite + 0.183 J/cm^2^	12.40	
*Y. enterocolitica*	0.4 J/cm^2^ + MAP (70% O_2_ and 30% N)	8.96	Reichel et al. (2020) [31]
	4.08 J/cm^2^ + MAP (70% O_2_ and 30% N)	15.79	
**Gram-Positive Bacteria**	Treatments *	**Reduction (%)**	**Reference**
*L. monocytogenes*	0.02 J/cm^2^ + NIR-H	24.91	Ha and Kang (2015) [20]
	0.04 J/cm^2^ + NIR-H	30.18	
	0.055 J/cm^2^ + NIR-H	31.45	
	0.07 J/cm^2^ + NIR-H	35.64	
	0.09 J/cm^2^ + NIR-H	44.55	
	0.11 J/cm^2^ + NIR-H	49.64	
	0.13 J/cm^2^ + NIR-H	62.36	
*L. innocua*	1 J/cm^2^ + FP (0.75 s)	55.57	Sommers et al. (2009) [34]
	1 J/cm^2^ + FP (1.5 s)	61.15	
	1 J/cm^2^ + FP (3 s)	63.41	
	2 J/cm^2^ + FP (0.75 s)	59.06	
	2 J/cm^2^ + FP (1.5 s)	60.45	
	2 J/cm^2^ + FP (3 s)	62.02	
	4 J/cm^2^ + FP (0.75 s)	56.10	
	4 J/cm^2^ + FP (1.5 s)	58.89	
	4 J/cm^2^ + FP (3 s)	67.77	
	VP + 1 J/cm^2^ + FP (1.5 s)	55.75	
	VP + 2 J/cm^2^ + FP (3 s)	55.75	
*L. monocytogenes* and *S. aureus*	0.5 J/cm^2^ + 5% LAE + VP	43.50	Sommers et al. (2010) [35]

* NIR-H—nir-infrared heating at 200.36 W/cm^2^/nm; LA—lactic acid; LAE—lauric-arginate ester; VP—vacuum packaging; PE—polyethylene; OPP—oriented polypropylene; ClearTite—ClearTite^®^ films; MAP—modified atmosphere packaging; FP—flash pasteurization.

**Table 4:** Physicochemical (lipid oxidation, lightness, redness, yellowness, total color difference, hardness, chewiness, cohesiveness, and springiness) parameters of meat products subjected to UV-C combined treatments.

Treatments *	LOX	PROTOX	L*/a*/b*/∆E	Shear Force (g)	Reference
0.01 J/cm^2^ + 6.5% LA	132.26	30.47	↓2.59/↓0.93/↓0.96/2.91	NA	Rosário et al. (2021) [33]
0.1 J/cm^2^ + 2% LA	100.00	35.19	↓1.19/↓2.18/↓3.10/3.97	NA	
0.1 J/cm^2^ + 11% LA	203.23	36.48	↓4.55/↓2.15/↓1.33/5.20	NA	
0.55 J/cm^2^ + 2% LA	232.26	14.16	↓2.15/↓2.47/↓2.04/3.86	NA	
0.55 J/cm^2^ + 11% LA	132.26	52.79	↓2.18/↓0.19/↓1.34/2.57	NA	
0.64 J/cm^2^ + 6.5% LA	258.06	41.63	↓1.52/↓3.21/↓1.05/3.70	NA	
0.33 J/cm^2^ + 0.1% LA	138.71	36.91	↓2.99/↓1.90/↓2.39/4.27	NA	
0.33 J/cm^2^ + 12.9% LA	164.52	25.32	↓2.69/↓2.44/↓2.56/4.44	NA	
0.33 J/cm^2^ + 6.5% LA	200.00	6.44	↓3.84/↓3.90/↓3.18/6.33	NA	
0.36 J/cm^2^ + 7.7% LA	190.32	5.58	NA	NA	
0.4 J/cm^2^ + MAP (70% O_2_ and 30% N_2_)	NA ^£^	NA	↓0.50/↓2.20/↓0.20/2.26	NA	Reichel et al. (2020) [31]
4.08 J/cm^2^ + MAP (70% O_2_ and 30% N_2_)	NA	NA	↑0.20/↓3.20/↑0.70/3.28	NA	
1 J/cm^2^ + FP (1.5 s)	NA	NA	↓7.49/↑0.20/↓0.49/7,51	24	Sommers et al. (2009) [34]
2 J/cm^2^ + FP (3 s)	NA	NA	↓6.88/↓2.07/↓0.79/7,23	122	
0.5 J/cm^2^ + 5% LAE	NA	NA	↓2.40/↓0.60/↓0.70/2.57	0.003	Sommers et al. (2010) [35]

Values are means obtained from the difference between each UV-C combined treatment and its control counterparts, with the effect indicated by symbols ↑ or ↓ (increase or decrease in relation to control), except for the total color difference (∆E). In this case, values indicate color changes in each specific treatment without comparison with the control. For instrumental texture parameters, values without symbols indicate that the difference from their control counterparts was insignificant. ^£^ NA—not analyzed; * LA—lactic acid; MAP—modified atmosphere packaging; FP—flash pasteurization; LAE—lauric-arginate ester. LOX (lipid oxidation) in %; PROTOX (protein oxidation) in %; L*—lightness; a*—redness; b*—yellowness; ∆E—total color difference.

## Data Availability

The data presented in this study are available on request from the corresponding author.
